# GAP-43 and BASP1 in Axon Regeneration: Implications for the Treatment of Neurodegenerative Diseases

**DOI:** 10.3389/fcell.2020.567537

**Published:** 2020-09-03

**Authors:** Daayun Chung, Andrew Shum, Gabriela Caraveo

**Affiliations:** Department of Neurology, Feinberg School of Medicine, Northwestern University, Chicago, IL, United States

**Keywords:** GAP-43, BASP1, phosphorylation, neural injury response, axon regeneration, neurodegenerative diseases

## Abstract

Growth-associated protein-43 (GAP-43) and brain acid-soluble protein 1 (BASP1) regulate actin dynamics and presynaptic vesicle cycling at axon terminals, thereby facilitating axonal growth, regeneration, and plasticity. These functions highly depend on changes in GAP-43 and BASP1 expression levels and post-translational modifications such as phosphorylation. Interestingly, examinations of GAP-43 and BASP1 in neurodegenerative diseases reveal alterations in their expression and phosphorylation profiles. This review provides an overview of the structural properties, regulations, and functions of GAP-43 and BASP1, highlighting their involvement in neural injury response and regeneration. By discussing GAP-43 and BASP1 in the context of neurodegenerative diseases, we also explore the therapeutic potential of modulating their activities to compensate for neuron loss in neurodegenerative diseases.

## Introduction

Axons integrate external cues to grow toward and arborize their terminals onto their correct targets. Such axonal behavior is critical for establishing proper connections, regenerating injured nerves, and retaining anatomical plasticity in adult brains (Skene, 1989). A group of growth-associated proteins (>100) (Costigan et al., 2002; Xiao et al., 2002), whose expression is upregulated during neuronal development and regeneration, mediates these functions (Jacobson et al., 1986; Widmer and Caroni, 1990; Mason, 2002). Growth-associated protein-43 (GAP-43) and brain acid-soluble protein 1 (BASP1) are two members of this group with many shared structural properties and functions (Mosevitsky, 2005). GAP-43 is solely expressed in the nervous system (Karns et al., 1987), whereas BASP1 is highly expressed in the nervous system and some non-neural tissues including the kidney and testis (Mosevitsky et al., 1997; Mosevitsky and Silicheva, 2011). Within neurons, GAP-43, and BASP1 are enriched in axon terminals (Meiri et al., 1986; Widmer and Caroni, 1990), where they regulate the actin cytoskeleton (Wiederkehr et al., 1997; Laux et al., 2000). By modulating actin dynamics, GAP-43, and BASP1 achieve their physiological functions in neurodevelopment, synaptic function, and nerve regeneration.

## Physiological Functions in the Nervous System

### Neurodevelopment

Growth-associated protein-43 and BASP1 are highly expressed during periods of active axon growth and synaptogenesis (McGuire et al., 1988; De La Monte et al., 1989; Widmer and Caroni, 1990). Their critical involvement in neurodevelopment has been demonstrated by gene knockout studies. Homozygous knockout of GAP-43 (GAP-43^–/–^) or BASP1 (BASP1^–/–^) leads to high neonatal lethality, resulting in 5–10% survival to adulthood (Strittmatter et al., 1995; Frey et al., 2000; Metz and Schwab, 2004). Surviving GAP-43^–/–^ animals exhibit defective pathfinding of retinal and commissural axons (Strittmatter et al., 1995; Shen et al., 2002), as well as an abnormal somatotopic map in the barrel cortex (Maier et al., 1999). In reflection of these anatomical defects, GAP-43^–/–^ animals demonstrate motor, sensory, and behavioral impairments (Metz and Schwab, 2004). Surviving BASP1^–/–^ animals also present evidence of impaired neurodevelopment including enlarged ventricles in the brain, axonal and synaptic abnormalities in the neocortex and hippocampus, and hyperactive behavior (Frey et al., 2000).

### Synaptic Function

As axons complete the innervation of their target areas, GAP-43 and BASP1 are downregulated in most brain regions. Interestingly, they remain highly expressed in areas of the adult brain implicated in learning and memory, including the neocortex and hippocampus (Benowitz et al., 1988; McGuire et al., 1988; Neve et al., 1988; Frey et al., 2000). In support of their importance in information storage, heterozygous GAP-43 knockout mice (GAP-43^+/–^) exhibit a selective impairment in contextual memory (Rekart et al., 2005). This phenotype can be explained by various synaptic functions of GAP-43. GAP-43 was shown to regulate endocytosis via its interaction with rabaptin-5, which functions in endocytic membrane fusion (Neve et al., 1998), and as a substrate of caspase-3, which mediates AMPA receptor endocytosis (Han et al., 2013). Also, antibodies against GAP-43 decreased the release of glutamate and noradrenaline (Dekker et al., 1989a; Hens et al., 1998), indicating its importance in neurotransmitter release. This function is thought to be mediated, at least in part, by its interaction with the presynaptic vesicle fusion complex (Syntaxin, SNAP-25, and VAMP) (Haruta et al., 1997). Moreover, GAP-43 was shown to enhance long-term potentiation (Routtenberg and Lovinger, 1985; Lovinger et al., 1986; Hulo et al., 2002). The presence of BASP1 on synaptic vesicles (Yamamoto et al., 1997) and its identification as a caspase-3 substrate (Han et al., 2013) suggest its potential role in synaptic vesicle cycling, which may have implications for neurotransmission, synaptic plasticity, and information storage.

### Nerve Regeneration

Another circumstance under which GAP-43 and BASP1 are upregulated is nerve regeneration following injury (Skene and Willard, 1981; Caroni et al., 1997). An increase in GAP-43 and BASP1 mRNA levels strongly correlates with enhanced regenerative capacity. This is evidenced by their robust upregulation in the regenerating dorsal root ganglion (DRG) axons following sciatic nerve injury but not in the non-regenerating DRG axons following dorsal rhizotomy (Mason, 2002). The upregulation of GAP-43 and BASP1 also correlated with axonal sprouting after stroke in the barrel cortex (Carmichael et al., 2005). Additionally, an increase in GAP-43 was associated with optogenetic-induced functional recovery from stroke in the primary motor cortex (Cheng et al., 2014). In a different rodent model of stroke, antisense oligonucleotides to GAP-43 abolished the enhancement of functional recovery induced by the basic fibroblast growth factor (Kawamata et al., 1999). These observations from rodent stroke models hint at the importance of GAP-43 in neuronal recovery following injury. More importantly, GAP-43 was shown to be essential for the regenerative response through knockdown studies. In adult rodents, climbing fibers retain high levels of GAP-43 and demonstrate structural plasticity after injury (Grasselli and Strata, 2013). Knockdown of GAP-43 in climbing fibers inhibited the sprouting of their axonal branches following laser-axotomy (Allegra Mascaro et al., 2013) and lesion of the inferior olive, where these fibers originate (Grasselli et al., 2011). Overexpression studies involving GAP-43 and BASP1 show these proteins are sufficient for f-actin accumulation and subsequent neurite formation in primary sensory neurons (Aigner and Caroni, 1995) and in Purkinje neurons (Buffo et al., 1997). Moreover, co-overexpression of GAP-43 and BASP1 was sufficient to drive the regeneration of DRG axons following spinal cord lesion in adult mice when peripheral nerve graft was provided (Bomze et al., 2001).

## Involvement in Neural Injury Response

In the central (CNS) and peripheral nervous systems (PNS), different external and internal factors lead to disparate outcomes of injury response. In the CNS, glial scar (Silver and Miller, 2004) and inhibitory glial factors such as myelin-associated glycoproteins and Nogo (Filbin, 2003) impede neuronal regeneration. Additionally, the inherent lack of axonal integrins and growth factor receptors limit the regrowth of neurons in the CNS (Koseki et al., 2017). As a result, positive outcomes of CNS injury remain limited to neuroprotection and marginal regenerative response. Neural injury stimulates neurons and glia to release cytokines and neurotrophic factors, which can activate growth-associated proteins such as GAP-43 and BASP1 to promote neuroprotection and regeneration.

### Cytokine Signaling

**FIGURE 1:**
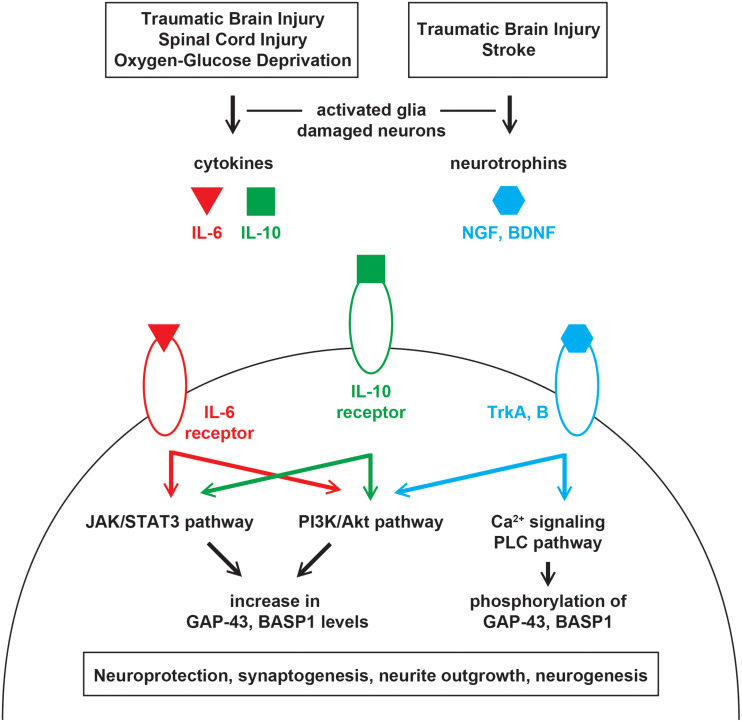
Injury-induced signaling pathways that regulate GAP-43 and BASP1. Injuries in the central nervous system leads to the release of cytokines and neurotrophins from damaged neurons and activated glia. Cytokines and neurotrophins bind their receptors on surviving neurons to activate various signaling cascades that regulate the expression level and phosphorylation of GAP-43 and BASP1. Alterations in GAP-43 and BASP1 levels and phosphorylation are associated with neuroprotection, synaptogenesis, neurite outgrowth, and neurogenesis.

Upon injury, leakage from damaged or dying cells leads to an excess of glutamate in the extracellular space (Bullock et al., 1998). This stimulates astrocytes, microglia, and neurons to secrete cytokines such as interleukin-6 (IL-6) and -10 (IL-10) (Morganti-Kossman et al., 1997; Acarin et al., 2000). Accordingly, IL-6 and -10 were found to be elevated in the cerebrospinal fluid and serum of patients with severe traumatic brain injury (TBI) (Kossmann et al., 1995; Csuka et al., 1999). After spinal cord injury, IL-6 treatment was shown to activate the JAK/STAT3 and PI3K/Akt pathways, upregulate GAP-43 and BASP1, and promote neurite outgrowth *in vitro* and synaptogenesis *in vivo* (Yang et al., 2012, 2015; Figure 1). The upregulation of GAP-43 and BASP1 was sensitive to the JAK2 inhibitor AG490 (Yang et al., 2015) but was not examined with a PI3K inhibitor. After oxygen-glucose deprivation, IL-10 treatment was shown to activate the JAK/STAT3 and PI3K/Akt pathways, upregulate GAP-43, and facilitate neuroprotection, neurite outgrowth, and synaptogenesis *in vitro* (Lin et al., 2015; Chen et al., 2016; Figure 1). The upregulation of GAP-43 was shown to be sensitive to the PI3K inhibitor LY294002 (Lin et al., 2015) but was not examined with a JAK2 inhibitor. The extent to which the neurite outgrowth observed *in vitro* translates into *in vivo* regeneration remains unclear.

### Neurotrophic Factor Signaling

Following injury, neurotrophic factors such as the nerve growth factor (NGF) (DeKosky et al., 1994; Chiaretti et al., 2009) and brain-derived neurotrophic factor (BDNF) (Miyake et al., 2002; Rostami et al., 2014) are upregulated in response to glutamate (Wetmore et al., 1994; Gwag et al., 1997) and cytokines (Kossmann et al., 1996). NGF and BDNF bind tropomyosin receptor kinase A and B, respectively, to initiate cell survival signaling via the PI3K/Akt pathway (Nguyen et al., 2010; Figure 1). In rodent models of stroke and TBI, the activation of NGF and BDNF signaling was shown to promote neuroprotection, synaptogenesis, and neurogenesis (Wu et al., 2008; Qi et al., 2014; Gudasheva et al., 2019). Such beneficial upregulation of NGF and BDNF was accompanied by an increase in GAP-43 levels when optogenetic stimulations were provided after stroke (Cheng et al., 2014). Given that BASP1 is similarly upregulated after stroke (Carmichael et al., 2005), its increase may also be mediated by NGF and BDNF. Moreover, GAP-43 was shown to be an essential effector of BDNF-driven neuroprotection (Gupta et al., 2009).

## Structural Properties and Domains

### Intrinsic Disorder and Phase Separation

Growth-associated protein-43 and BASP1 are acidic proteins with isoelectric points of 4.4–4.6 (Mosevitsky et al., 1994). Their molecular weights are 23–25 kDa, but they appear at higher molecular weights on SDS-PAGE (Mosevitsky et al., 1994). These proteins are enriched in alanine (22% in GAP-43, 21% in BASP1) and proline (8% in GAP-43, 12% in BASP1), which gives rise to a high content of type II polyproline helix (32 ± 5% in GAP-43, 37 ± 2% in BASP1) characteristic of intrinsically disordered proteins (Forsova and Zakharov, 2016). Nuclear magnetic resonance spectroscopy also indicates that GAP-43 and BASP1 have unordered structures (Zhang et al., 1994; Geist et al., 2013). Intrinsically disordered proteins lack a stable three-dimensional structure and have the ability to engage in multivalent interactions (Haynes et al., 2006). Through multivalent interactions, intrinsically disordered proteins undergo liquid-liquid phase separation and form membraneless compartments that facilitate their functions (Li et al., 2012). Many proteins involved in actin assembly have intrinsically disordered regions, and phase separation mediated by these regions underlies their regulation of actin dynamics (Sun et al., 2017; Miao et al., 2018). Moreover, phosphorylation of intrinsically disordered proteins affects phase separation by modulating their charge and electrostatic interactions (Aumiller and Keating, 2016; Miao et al., 2018). Given that GAP-43 and BASP1 are phosphoproteins, they may transmit signals from kinases and phosphatases to the actin cytoskeleton via phase separation.

### PEST Sequence and High Turnover Rate

Growth-associated protein-43 and BASP1 have peptide sequences rich in proline, glutamate, serine, and threonine (PEST) (Barnes and Gomes, 1995; Mosevitsky et al., 1997). While PEST regions vary in their sequences and lengths, they all serve as signals for rapid proteolysis (Rechsteiner and Rogers, 1996). The presence of PEST sequences indicates that GAP-43 and BASP1 are short-lived proteins, however, this has yet to be experimentally verified.

### Effector Domain and CaM Binding

**FIGURE 2:**
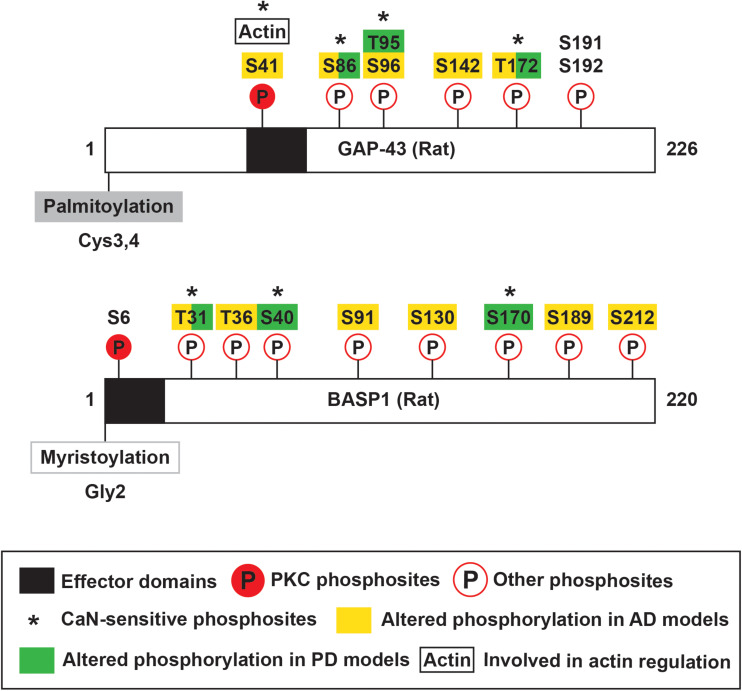
Schematic of rat GAP-43 and BASP1 structure and post-translational modifications. GAP-43 and BASP1 are characterized by N-terminal fatty acylation, effector domains, and multiple phosphosites. GAP-43 is palmitoylated at Cysteine-3 and -4, and BASP1 is myristoylated at the N-terminal end. Their effector domains enable calmodulin binding and are subjected to phosphorylation by PKC. Phosphorylation of GAP-43 by PKC regulates various functions including actin cytoskeleton dynamics. GAP-43 and BASP1 are also regulated by other kinases and phosphatases, and such post-translational modifications are implicated in Alzheimer’s and Parkinson’s Diseases.

Growth-associated protein-43 and BASP1 have regions termed the effector domain (ED) that are enriched in basic and hydrophobic residues, bind calmodulin (CaM), and are phosphorylated by protein kinase C (PKC) (Cimler et al., 1985; Apel et al., 1990; Maekawa et al., 1993). The basic and hydrophobic residues in the ED also contribute to membrane association of these proteins (O’Neil and DeGrado, 1990; Mosevitsky et al., 1997; Mosevitsky, 2005). GAP-43 ED consists of residues 37–52 (KIQASFRGHITRKKLK) (Mosevitsky, 2005; Figure 2), which includes a canonical CaM-binding site termed the IQ motif (IQxxxRGxxxR) (Bähler and Rhoads, 2002). GAP-43 was shown to bind CaM with higher affinity in the absence of or at low Ca^2+^ and to dissociate at high Ca^2+^ (Andreasen et al., 1983; Alexander et al., 1987; Gamby et al., 1996). Hence, GAP-43 has been proposed to accumulate CaM at specific sites and release them upon local Ca^2+^ elevation to sharpen the downstream response (Alexander et al., 1987; Mosevitsky, 2005). Unlike GAP-43, BASP1 ED is located at the N-terminal end (Myristoylation-GGKLSKKKKGY) (Mosevitsky, 2005; Figure 2). BASP1 lacks an IQ motif and instead binds CaM through alternating basic and hydrophobic residues (Takasaki et al., 1999). These alternating parts include the myristoyl moiety, which passes through a tunnel formed by hydrophobic pockets in the N- and C-terminal domains of CaM (Takasaki et al., 1999; Matsubara et al., 2004). BASP1 was shown to bind CaM with stronger affinity than GAP-43 (Maekawa et al., 1994) and in the presence of Ca^2+^ (Maekawa et al., 1993), suggesting a different mode of action than GAP-43.

### Formation of Oligomers

Growth-associated protein-43 and BASP1 form oligomers in the presence of anionic phospholipids or sodium dodecyl sulfate (SDS) (Zakharov and Mosevitsky, 2010). Among anionic phospholipids, phosphatidylinositol 4,5-bisphosphate (PI(4,5)P_2_) was the most potent driver of oligomerization (Zakharov and Mosevitsky, 2010). Interestingly, GAP-43 and BASP1 accumulate PI(4,5)P_2_ on the inner surface of the plasma membrane, and this clustering is important for initiating signaling cascades that regulate the actin cytoskeleton (Laux et al., 2000). The role of PI(4,5)P_2_ in driving oligomerization suggests that PI(4,5)P_2_-induced oligomers may be important for this process. Oligomerization of GAP-43 and BASP1 results in α-helix formation within their EDs while preserving the overall structural disorder (Forsova and Zakharov, 2016). A significant level of disorder in oligomers has been proposed to enhance the flexibility of interactions and to enable the reversion to monomers (Tompa and Fuxreiter, 2008). In support of the flexibility in binding, GAP-43 and BASP1 were observed to form heterooligomers of different stoichiometries *in vitro* and in presynaptic membranes (Forsova and Zakharov, 2016). In agreement with the reversibility, GAP-43 and BASP1 oligomers were shown to dissociate into monomers upon removal of SDS or binding of CaM (Zakharov and Mosevitsky, 2010).

## Transcriptional and Post-Transcriptional Regulation

In developing and regenerating neurons, the levels of GAP-43 and BASP1 mRNAs and proteins are upregulated (Widmer and Caroni, 1990; Perrone-Bizzozero et al., 1991; Mason, 2002). Studies present conflicting findings regarding the contributions of transcriptional activation to increased GAP-43 levels. The GAP-43 gene has two promoters, distal (P1) and proximal (P2), that are highly conserved between the rat and human genes (Eggen et al., 1994; de Groen et al., 1995). P1 contains classical promoter elements including TATA and CCAAT boxes (Nedivi et al., 1992), as well as a repressive element shown to inhibit GAP-43 expression in non-neuronal cells (Weber and Skene, 1997). P2 lacks classical promoter elements but contains a conserved enhancer box (E1) that can bind basic helix-loop-helix transcription factors (Chiaramello et al., 1996), which have critical roles in neural cell fate specification and differentiation (Dennis et al., 2019). In zebrafish, a 1 kb fragment spanning P1 and P2 of the rat GAP-43 gene was shown to developmentally regulate the expression of a downstream transgene in neurons (Udvadia et al., 2001). In contrast to this finding, developing rat cortical neurons and nerve growth factor-induced PC12 cells showed no change in GAP-43 pre-mRNA levels despite an increase in GAP-43 mRNA levels (Perrone-Bizzozero et al., 1991). This finding indicates that mRNA stability mainly contributes to the observed upregulation of GAP-43. The stabilization of GAP-43 mRNA is dependent on the highly conserved 3′ untranslated region (Kohn et al., 1996; Tsai et al., 1997), where the neural-specific RNA-binding protein HuD binds (Chung et al., 1997). The expression of HuD and GAP-43 are concomitantly increased during neuritogenesis (Anderson et al., 2001), in regenerating nerves (Anderson et al., 2003), and following spatial learning in rodent hippocampi (Quattrone et al., 2001; Pascale et al., 2004). Based on these observations, HuD was hypothesized to stabilize GAP-43 mRNA under physiological conditions. In support of this hypothesis, transgenic mice overexpressing HuD exhibited an increase in GAP-43 mRNA but not pre-mRNA (Bolognani et al., 2006). Also, the half-life of GAP-43 mRNA from these transgenic mice was significantly longer than those from non-transgenic controls (Bolognani et al., 2006). The HuD-dependent stabilization of GAP-43 mRNA is positively regulated by PKC (Perrone-Bizzozero et al., 1993; Sanna et al., 2014) and is inhibited by the KH-type splicing regulatory protein, which competes with HuD to bind and promote the degradation of GAP-43 mRNA (Bird et al., 2013). Compared to GAP-43, little is known about the transcriptional and post-transcriptional control of BASP1. In the chicken BASP1 gene, a 135 bp region in the 5′ end of exon 1 was shown to bind the transcription factors Sp1 and Myc (Hartl et al., 2009). This regulatory region was sufficient to activate transcription and to mediate Myc-induced suppression of BASP1 (Hartl et al., 2009). Additionally, post-transcriptional regulation of BASP1 by its processed pseudogene has been proposed but not experimentally verified (Uzumcu et al., 2009).

## Post-Translational Modifications

### Fatty Acylation

**FIGURE 3:**
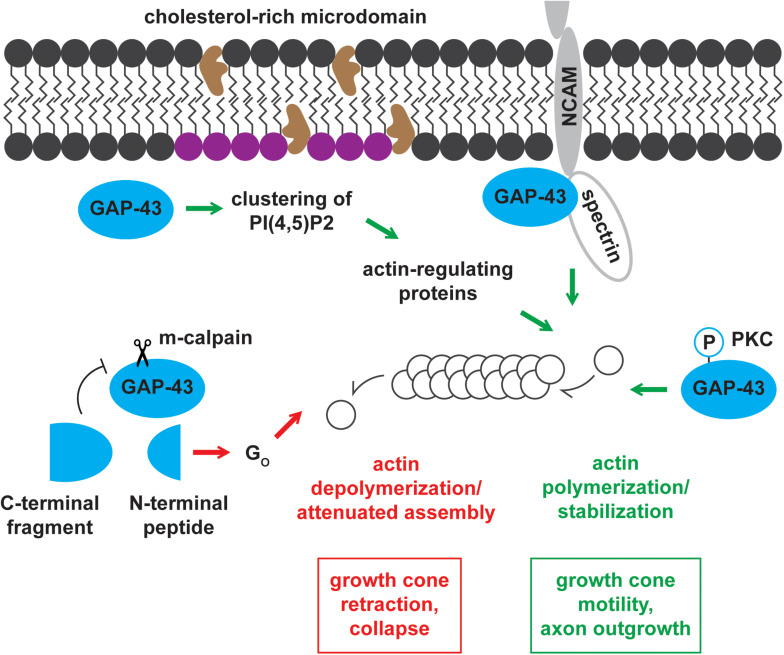
Mechanisms of actin cytoskeleton regulation by GAP-43. GAP-43 positively and negatively regulates the actin cytoskeleton through multiple routes of action. GAP-43 promotes growth cone motility and axon outgrowth by (1) recruiting actin-regulating proteins by forming PI(4,5)P2 clusters, (2) interacting with NCAM and spectrin, and (3) stabilizing actin filaments in a PKC-dependent manner. Conversely, m-calpain cleavage product of GAP-43 drives growth cone retraction and collapse through the G_*o*_ signaling pathway.

Growth-associated protein-43 and BASP1 mainly localize to membranes (Skene et al., 1986; Maekawa et al., 1993), and their membrane associations are partially mediated by fatty acylation. GAP-43 is post-translationally palmitoylated at Cysteine-3 and -4 (Skene and Virág, 1989; Figure 2). Its palmitoylation can occur in the endoplasmic reticulum-Golgi intermediate compartment (ERGIC), Golgi apparatus, and plasma membrane (McLaughlin and Denny, 1999). Upon palmitoylation, GAP-43 can be sorted to the tips of growing neurites (Gauthier-Kemper et al., 2014). Palmitoylation of GAP-43 is dynamically regulated, as suggested by the low percentage (∼35%) of fatty acylated GAP-43 at steady state in PC12 and COS-1 cells (Liang et al., 2002). The dynamic regulation of palmitoylation affects GAP-43 functions. Changes in palmitoylation enable GAP-43 to cycle between pathways independent of and involving G_o_, a heterotrimeric GTP-binding protein enriched in growth cones (Edmonds et al., 1990). N-terminal peptides of GAP-43 produced by the Ca^2+^-dependent protease m-calpain interact with and activate G_o_ signaling cascade that leads to growth cone collapse (Strittmatter et al., 1994; Zakharov and Mosevitsky, 2007; Figure 3). Palmitoylation reduces the ability of the N-terminal peptides to stimulate G_o_, thereby blocking G_o_ signaling-induced growth cone collapse (Sudo et al., 1992). Additionally, palmitoylation appears to be important for the switch from promoting axon growth to stabilizing synapses upon successful target innervation (Patterson and Skene, 1999). Experimental evidence shows that palmitoylation of GAP-43, when inhibited, reversibly stalls neurite outgrowth (Hess et al., 1993) and is significantly reduced at the early phase of synapse maturation (Patterson and Skene, 1999). Unlike GAP-43, BASP1 is co-translationally myristoylated at the N-terminal end (Mosevitsky et al., 1997; Takasaki et al., 1999; Figure 2). In the rat brain, BASP1 molecules and N-terminal fragments appear predominantly in the myristoylated form (Mosevitsky et al., 1997; Zakharov et al., 2003). Myristoylation of BASP1 was shown to promote membrane association and to enable CaM binding (Takasaki et al., 1999; Matsubara et al., 2004).

### Phosphorylation

Growth-associated protein-43 and BASP1 EDs undergo Ca^2+^-dependent phosphorylation by PKC. PKC phosphosites in GAP-43 and BASP1 are Serine-41 and Serine-6, respectively (Apel et al., 1990; Maekawa et al., 1994; Figure 2). GAP-43 Serine-41 can also be dephosphorylated by the Ca^2+^/CaM-dependent phosphatase calcineurin (CaN) (Liu and Storm, 1989). Phosphorylation by PKC abolishes CaM binding to both GAP-43 and BASP1 (Apel et al., 1990; Takasaki et al., 1999). More importantly, PKC-mediated phosphorylation has significant functional consequences. Phosphorylated GAP-43 not only promotes actin polymerization and stabilization (He et al., 1997; Korshunova et al., 2007) but also interacts with presynaptic vesicle fusion complex (Syntaxin, SNAP-25, and VAMP) (Haruta et al., 1997). Through these molecular pathways, phosphorylated GAP-43 facilitates axon guidance (Dent and Meiri, 1998), axon outgrowth (Aigner et al., 1995; Korshunova et al., 2007), neurotransmission (Dekker et al., 1989b; Heemskerk et al., 1990), and synaptic plasticity (Routtenberg and Lovinger, 1985; Lovinger et al., 1986; Hulo et al., 2002). The modulation of BASP1 functions by PKC remains to be studied. In addition to PKC phosphosites, many residues of GAP-43 and BASP1 were found to be phosphorylated. To name a few, GAP-43 was phosphorylated at Serine-96 and Threonine-172 by unknown kinase(s) (Spencer et al., 1992), Serine-191 and -192 by Casein Kinase II (Apel et al., 1991), and Serine-96 by c-Jun N-terminal kinase (JNK) (Kawasaki et al., 2018; Figure 2). Additional phosphosites on GAP-43 (Serine-86, Threonine-95, and Threonine-172) and BASP1 (Threonine-31, Serine-40, Serine-170) were found to be sensitive to CaN (Caraveo et al., 2017; Figure 2). The phosphorylation of GAP-43 and BASP1 at these other residues may be of functional significance. For example, JNK phosphorylates GAP-43 in growth cone membranes, and this modification is associated with axon growth and regeneration (Kawasaki et al., 2018).

## Mechanisms of Actin Cytoskeleton Regulation

Regulation of the actin cytoskeleton is important for axon guidance and growth (Gomez and Letourneau, 2014; Blanquie and Bradke, 2018), endocytosis (Smythe and Ayscough, 2006), and exocytosis (Eitzen, 2003). Therefore, understanding how GAP-43 and BASP1 modulate actin dynamics provides insight into the mechanisms through which they achieve their physiological functions. To test if GAP-43 and BASP1 perform their functions through identical pathways, knockin mice expressing GAP-43 in place of BASP1 was generated (Frey et al., 2000). While these mice exhibited no gross abnormality in the brain, they showed small morphological differences in axon sprouts (Frey et al., 2000). This indicates that GAP-43 and BASP1 act through partially redundant pathways, a concept that was reinforced by observations of phenotypic differences between GAP-43- and BASP1-induced neurite outgrowth and of synergistic axon sprouting with co-overexpression of GAP-43 and BASP1 (Caroni et al., 1997).

### Signaling Through PI(4,5)P_2_ Clusters

Growth-associated protein-43 and BASP1 regulate actin dynamics through a shared pathway involving PI(4,5)P_2_ (Laux et al., 2000; Caroni, 2001). In the intracellular surface of cholesterol-rich rafts, GAP-43 and BASP1 co-distribute with and promote the clustering of PI(4,5)P_2_ (Laux et al., 2000). Concentrating PI(4,5)P_2_ in local environments is thought to enhance the recruitment of actin-regulating proteins (Caroni, 2001). These proteins include WASP and ERM proteins known for their roles in actin polymerization and actin cytoskeleton-membrane crosslinking, respectively (Sechi and Wehland, 2000). The functions of WASP and ERM proteins contribute to growth cone motility and outgrowth (Figure 3).

### PKC-Dependent Modulation of Actin

Growth-associated protein-43 binds and directly regulates actin filaments in a PKC-dependent manner (He et al., 1997). Phosphorylated GAP-43 binds actin with higher affinity (K_d_ = 161 nM) compared to the unphosphorylated form (K_d_ = 1.2 uM) (He et al., 1997). In accordance with this enhanced binding, the cytoskeletal association of GAP-43 increases with phosphorylation (Tejero-Díez et al., 2000). Moreover, phosphorylated GAP-43 stabilizes actin filaments, thereby promoting growth cone extension and motility (He et al., 1997; Figure 3). On the other hand, unphosphorylated GAP-43 inhibits growth cone extension likely by serving as a barbed end-capping protein (He et al., 1997). Capping proteins significantly increase the concentration of actin monomers needed for polymerization and attenuate actin assembly, which is critical for growth cone extension. While BASP1 may similarly regulate the actin cytoskeleton, this hypothesis remains to be tested.

### Functional Association With NCAM-180 and Spectrin

Growth-associated protein-43 has also been proposed to modulate actin dynamics through the functional association with the neural cell adhesion molecule-180 (NCAM-180) and the cytoskeletal protein spectrin (Korshunova et al., 2007; Figure 3). NCAMs engage in homophilic and heterophilic binding with components of other cells and the extracellular matrix (ECM) (Ditlevsen et al., 2008). These interactions, in addition to establishing cell-cell and cell-ECM adhesions, initiate intracellular signaling cascades important for neuronal development, synaptic plasticity, and regeneration (Ditlevsen et al., 2008). NCAMs directly associate with intracellular signaling molecules to activate these downstream cascades (Ditlevsen et al., 2008). NCAM-180 has been shown to mediate neurite outgrowth by interacting with spectrin (Leshchyns’ka et al., 2003), and spectrin was shown to bind GAP-43 (Riederer and Routtenberg, 1999). Based on these observations, NCAM-180, spectrin, and GAP-43 have been hypothesized to function in a complex to mediate neurite outgrowth. In support of this hypothesis, NCAM-mediated neurite outgrowth, in the presence of GAP-43, required functional NCAM-180 and spectrin (Korshunova et al., 2007). BASP1 was shown to function independently of this mechanism in PC12 cells and hippocampal neurons, where BASP1 failed to substitute the stimulation of NCAM-mediated neurite outgrowth by GAP-43 (Korshunova et al., 2008).

## Properties of GAP-43 and BASP1 and Their Link to Neurodegenerative Diseases

### Intrinsic Disorder and Phase Separation in Pathological Protein Aggregation

Growth-associated protein-43 and BASP1 are intrinsically disordered proteins (Zhang et al., 1994; Geist et al., 2013; Forsova and Zakharov, 2016). The functional significance of this property remains understudied in both physiological and pathological contexts. In many neurodegenerative diseases, intrinsically disordered proteins form soluble and fibril aggregates that are central to the pathogenesis (Chiti and Dobson, 2006). The recent discovery of liquid-liquid phase separation, which condensates disordered proteins, gained interest as a potential mechanism of pathological protein aggregation (Elbaum-Garfinkle, 2019). Furthermore, proteins forming aggregates in neurodegenerative diseases such as Tau (Wegmann et al., 2018) and Huntingtin (Peskett et al., 2018) were shown to undergo phase separation. The involvement of disordered proteins in neurodegenerative diseases questions whether GAP-43 and BASP1 also phase separate in this context, whether this process turns aberrant, and what the functional consequences are.

### Post-transcriptional Regulation in Neurodegenerative Diseases

Post-transcriptional regulation allows a rapid adjustment of the location and level of protein expression (Bronicki and Jasmin, 2013). This spatiotemporal regulation is particularly important in neurons because of their large and complex morphology (Bronicki and Jasmin, 2013). A key regulator of this process is the neuron-specific RNA-binding protein HuD (Bronicki and Jasmin, 2013). HuD post-transcriptionally regulates GAP-43 during neuritogenesis, regeneration, and learning (Anderson et al., 2001, 2003; Quattrone et al., 2001; Pascale et al., 2004). Interestingly, HuD is associated with various neurodegenerative disorders. The level of HuD was shown to be decreased in the hippocampus of Alzheimer’s Disease patients (Amadio et al., 2009). This decrease correlated with diminished expression of ADAM10, a protein known to reduce the generation of pathogenic amyloid-β peptides (Amadio et al., 2009). From genetic studies, HuD was also identified as a susceptibility gene for Parkinson’s Disease (Noureddine et al., 2005; DeStefano et al., 2008). Additionally, HuD was shown to aberrantly interact with FUS mutant that is causally linked to Amyotrophic Lateral Sclerosis (De Santis et al., 2019). In light of these findings, the post-transcriptional regulation of GAP-43 may be dysregulated in neurodegenerative disorders.

### PKC and CaM in Neurodegenerative Diseases

Growth-associated protein-43 and BASP1 engage in intracellular Ca^2+^ signaling through PKC and CaM (Cimler et al., 1985; Apel et al., 1990; Maekawa et al., 1993). Ca^2+^ signaling plays a central role in neuronal physiology, and Ca^2+^ dyshomeostasis contributes to the pathogenesis of neurodegenerative disorders (Bezprozvanny, 2009). As part of Ca^2+^ signaling cascades, PKC and CaM significantly impact the disease states.

Protein kinase C has a vital role in memory encoding and storage (Sun and Alkon, 2014); therefore, its involvement in Alzheimer’s Disease – a major form of dementia – has been studied extensively. Few studies showed that the activity of PKC is decreased in the brains of Alzheimer’s Disease patients (Wang et al., 1994; Matsushima et al., 1996), suggesting potential adverse effects of its diminished activity. In alignment with this finding, the PKC activator bryostatin reduced the production of amyloid-β peptides and premature mortality in mouse models of Alzheimer’s Disease (Etcheberrigaray et al., 2004). On the other hand, whole genome-sequencing of late-onset Alzheimer’s Disease patients identified gain-of-function mutations in PKC (Alfonso et al., 2016). This study demonstrated that enhanced PKC activity mediates synaptic depression induced by amyloid-β peptides (Alfonso et al., 2016). Other studies also linked PKC activation to synaptic changes such as reduced cell-surface expression of AMPA receptor (Liu et al., 2010) and dysregulated structural plasticity (Calabrese and Halpain, 2005). These findings point to the importance of balanced PKC activity in neuronal physiology. They also raise the possibility that PKC-dependent functions of GAP-43 and BASP1 affect cellular pathways whose dyshomeostasis contribute to neurodegenerative diseases.

Calmodulin connects Ca^2+^ signals to cellular functions through various effector proteins (Chin and Means, 2000). These effectors include Ca^2+^/CaM-dependent kinases and phosphatases like CaMKII and CaN. CaMKII and CaN critically regulate synaptic functions (Mulkey et al., 1994; Pang et al., 2010) and contribute to synaptopathy in neurodegenerative diseases (Kuchibhotla et al., 2008; Gu et al., 2009; Teravskis et al., 2018). CaM also affects pathogenic processes such as Ca^2+^ dysregulation and pathogenic protein fibrillation by directly binding the plasma membrane Ca^2+^ APTase (PCMA) and amyloid-β peptides (Berrocal et al., 2012; Corbacho et al., 2017). Likewise, CaM binding by GAP-43 and BASP1 may directly and indirectly affect processes involved in neurodegenerative disorders.

## Functions of GAP-43 and BASP1 and Their Link to Neurodegenerative Diseases

Growth-associated protein-43 and BASP1 regulate the actin cytoskeleton, which in turn modulates axon outgrowth (Gomez and Letourneau, 2014; Blanquie and Bradke, 2018) and synaptic functions (Eitzen, 2003; Smythe and Ayscough, 2006). Actin dynamics are altered in neurodegenerative disorders, and this change culminates in structural defects and synaptic dysfunctions. Disruption of dendritic actin filaments were observed in *Drosophila* models of polyglutamine diseases (Lee et al., 2011). This disruption was associated with decreased dendritic complexity – an early deficit that may contribute to the pathogenesis (Lee et al., 2011). Similarly, decreased levels of synaptosomal actin filaments were detected in mouse models and patient brains of Alzheimer’s Disease (Kommaddi et al., 2018). This reduction inversely correlated with dendritic spine density and behavioral performance (Kommaddi et al., 2018). Moreover, in *Drosophila*, pathogenic tau mutant and amyloid-β promoted abnormal accumulation and bundling of actin filaments, which correlated with neurotoxicity (Fulga et al., 2007).

In addition, actin binding proteins contribute to the pathogenesis of neurodegenerative disorders. For instance, the actin depolymerizing factor cofilin was found to be hyperactivated in Alzheimer’s Disease (Bamburg et al., 2010). The accumulation of hyperactivated cofilin formed cofilin-actin rods that led to impaired synaptic functions and synapse loss (Bamburg et al., 2010; Cichon et al., 2012; Munsie and Truant, 2012). Conversely, the level of the actin stabilizing protein drebrin was dramatically reduced in Alzheimer’s Disease (Harigaya et al., 1996; Hatanpää et al., 1999). A reduction in drebrin levels impaired neuritogenesis (Geraldo et al., 2008), resulted in synaptic dysfunctions (Kojima and Shirao, 2007), and correlated with cognitive impairment (Counts et al., 2006). These findings warrant examination of the actin regulators GAP-43 and BASP1 in neurodegenerative diseases.

## Regulation of GAP-43 and BASP1 in Neurodegenerative Diseases

Growth-associated protein-43 and BASP1 were examined in many neurodegenerative diseases – Alzheimer’s Disease (Masliah et al., 1991; De La Monte et al., 1995; Bogdanovic et al., 2000; Rekart et al., 2004; Musunuri et al., 2014; Tagawa et al., 2015), Parkinson’s Disease (Caraveo et al., 2017; Saal et al., 2017; Wang et al., 2019b), Huntington’s Disease (Apostol et al., 2006; Dong and Cong, 2018), Amyotrophic Lateral Sclerosis (Parhad et al., 1992; Ikemoto et al., 1999; Andrés-Benito et al., 2017), and Spinal Muscular Atrophy (Fallini et al., 2016) – through studies of human patients and cellular and animal models. Most of these studies present correlational changes in GAP-43 and BASP1 but do not address their functional implications. In the following sections, GAP-43 and BASP1 will be discussed in the context of Alzheimer’s and Parkinson’s Disease, two neurodegenerative diseases in which they have been studied the most.

### Alzheimer’s Disease

Alzheimer’s Disease (AD) is the most common neurodegenerative disorder causing dementia (Barker et al., 2002). AD is neuropathologically characterized by extracellular amyloid beta (Aβ) deposits termed amyloid plaques (Masters et al., 1985) and intracellular neurofibrillary tangles composed of the microtubule-binding protein tau (Goedert et al., 1988). While AD involves a widespread loss of neurons, it primarily affects cholinergic neurons in the basal forebrain (Whitehouse et al., 1982), noradrenergic neurons in the locus coeruleus (Bondareff et al., 1982), and pyramidal neurons in the entorhinal cortex, subiculum, and hippocampal CA1 (Hyman et al., 1984; Morrison and Hof, 2002). These vulnerable neurons have long and thin axons with sparse myelination (Braak and Del Trecidi, 2015). Such axonal properties increase the energetic demand and exposure to pathogenic species that contribute to the vulnerability in AD (Braak and Del Trecidi, 2015). Immunohistochemical studies found that GAP-43 levels were decreased in the neocortex but was preserved or even elevated in the hippocampus of AD patients (Masliah et al., 1991; De La Monte et al., 1995; Bogdanovic et al., 2000; Rekart et al., 2004). The reduction in cortical GAP-43 immunoreactivity likely reflects a profound neuron loss. The preservation or increase in hippocampal GAP-43 immunoreactivity raises the possibility that surviving neurons, by upregulating GAP-43, initiate axon outgrowth to compensate for the lost connections. Hippocampal GAP-43 immunoreactivity was observed in dystrophic neurites associated with plaques and correlated with aberrant sprouting (Masliah et al., 1991; Bogdanovic et al., 2000), which is characteristic of synaptic pathology in AD (Masliah, 1995). Based on this observation, GAP-43-associated axon outgrowth appears to be unsuccessful in establishing functional connections and seems to be contributing to the synaptic pathology instead. Examination of entorhinal fibers in amyloid precursor protein transgenic mice provides evidence that this aberrant sprouting is driven by amyloid deposition (Phinney et al., 1999). This finding highlights the importance of extrinsic factors on the successful regenerative response and emphasizes the need for combined therapy that overcomes both extrinsic and intrinsic barriers to axon remodeling. In agreement with the immunohistochemical data, quantitative mass spectrometry detected a reduction of GAP-43 in the temporal neocortex of AD patients (Musunuri et al., 2014). The same study also measured decreased levels of BASP1 in AD patients (Musunuri et al., 2014). In addition to their changes in expression, the extent of their phosphorylation was altered in AD. The overall phosphorylation of both proteins decreased in the temporal lobe of AD patients compared to non-AD individuals (Tagawa et al., 2015). For a detailed analysis of the phosphoproteome over the course of disease progression, four mouse models of AD at early, middle, and late time points were examined with respect to control mice. This analysis identified 5 phosphosites in GAP-43 and 6 phosphosites in BASP1 (Figure 2 shows equivalent sites in rat proteins based on sequence alignment in Clustal Omega; Tagawa et al., 2015). Phosphorylation at these sites generally increased in the middle stage of pathology and progressively decreased (Tagawa et al., 2015). Although the physiological relevance of these changes remains to be examined, this study prompts us to explore the possibility of modulating GAP-43 and BASP1 phosphorylation for therapeutic interventions in AD.

### Parkinson’s Disease

Parkinson’s Disease (PD) is the most common neurodegenerative movement disorder (Tysnes and Storstein, 2017). PD is neuropathologically characterized by the accumulation of α-synuclein inclusions termed Lewy bodies (Spillantini et al., 1997). In PD, dopaminergic neurons in the substantia nigra pars compacta (SNc) primarily degenerate, causing motor symptoms such as bradykinesia, rigidity, and tremor (Tysnes and Storstein, 2017). The axons of the SNc dopaminergic neurons are long, thin, and poorly myelinated (Braak et al., 2004). Additionally, they branch extensively in the striatum and form extraordinarily large numbers of synapses (Bolam and Pissadaki, 2012) with transmitter release sites numbering up to 300,000 (Matsuda et al., 2009). These axonal properties contribute to the selective vulnerability in PD (Braak et al., 2004). A transcriptome-based meta-analysis of multiple studies found that GAP-43 and BASP1 are downregulated in the brains of PD patients. This same study identified BASP1 as an important regulator of other differentially expressed genes associated with synaptic signaling. Immunoreactivity of tyrosine hydroxylase (TH), a marker for dopaminergic neurons, was reduced in the SNc and striatum of PD patients in reflection of a substantial neuron loss (Saal et al., 2017). However, in the remaining TH+ SNc dopaminergic neurons, GAP-43 protein and mRNA levels were likewise decreased. In agreement with these data, reduced GAP-43 expression has also been detected in the cerebral spinal fluid of PD patients (Sjogren et al., 2000). Further evidence implicates the involvement of GAP-43 and BASP1 in PD. In an *in vitro* scratch lesion model using α-syn mutations causing autosomal-dominant forms of PD, the study found reduced neurite regeneration and subsequent loss of dopaminergic neurons accompanied by a reduction of striatal expression of GAP-43 (Tonges et al., 2014). In one PD patient, infusion with glial cell-derived neurotrophic factor at the putamen provided benefits even after cessation of treatment (Patel et al., 2013). While these reports suggest that increasing GAP-43 expression and therefore the axonal tree would be beneficial, other groups suggest that reduction of the axonal tree, in fact, confers protection in models of PD (Pacelli et al., 2015). The apparent discrepancy between these studies can be addressed if taken into consideration that the effect in neuronal sprouting needs to be regulated. In support of this idea, one study found that modulation of the activity of GAP-43 and BASP1 through CaN can alter the degeneration of axonal trees and confer neuroprotection in a rat model of PD (Caraveo et al., 2017). This model displayed presynaptic and behavioral impairments along with hypophosphorylation at 3 CaN-sensitive sites each in GAP-43 and BASP1 (Figure 2; Caraveo et al., 2017). Treatment with low doses of Tacrolimus, which partially inhibits CaN, ameliorated the presynaptic and behavioral deficits in addition to rescuing phosphorylation at these sites (Caraveo et al., 2017). This correlation suggests a potential involvement of GAP-43 and BASP1 phosphorylation in PD pathogenesis. Interestingly, Tacrolimus is an FDA-approved drug currently in widespread clinical use at high doses to suppress the rejection of organs in transplant patients, a process in which CaN also plays a critical role (Tron et al., 2019). This opens the possibility that Tacrolimus could be repurposed as a potential therapy for the treatment of PD.

## Concluding Remarks

Growth-associated protein-43 and BASP1 are essential for developing axons to grow toward their correct targets and form synaptic connections during neuronal development and after neural injury. These axonal functions highly depend on their expression levels and phosphorylation status, which when modulated appropriately, can stimulate mature neurons to re- enter a growth state. This transition, which can protect and enable surviving neurons to re-establish functional connections, has been explored as a therapeutic avenue for neurodegenerative diseases. Specifically, the effects of delivering agents that upregulate GAP-43, such as BDNF, have been tested in animal models of PD (Gupta et al., 2009). BDNF treatment, although unable to revert neurodegeneration, demonstrated protective effects on remaining neurons and ameliorated behavioral impairments (Palasz et al., 2020). Several investigations into understanding the mechanism of potential therapies have identified GAP-43 as either upregulated or essential for its ameliorative effects. Levetiracetam for treatment of retinopathy (Mohammad et al., 2019) as well as for the repair of convulsant- induced cognitive impairment (Wang et al., 2019a) directly signals through the PKC/GAP-43 signaling pathway. Similarly, TGN-020 for treatment of spinal cord injury (Li et al., 2019b), and senegenin for the potential treatment for Aβ-induced neurotoxicity (Jesky and Chen, 2016), involve upregulation of GAP-43 protein levels for both neuroprotection and *in vitro* regeneration. Low GAP-43 levels in cerebrospinal fluid were associated with a poorer response to treatment of primary progressive multiple sclerosis using fingolimod or alemtuzumab (Sandelius et al., 2019) suggesting that GAP-43 is important in mediating its therapeutic effects. In rat models of PD, treatments with Pilose antler extracts led to an increase in striatal GAP-43 protein expression and less dopaminergic SNc neuronal cell death (Li et al., 2019a). Despite the positive effects of neurotrophic factors in animal models, their short half-life, low bioavailability, and limited permeability through the blood-brain barrier (BBB) imposed challenges in their application to patients (Palasz et al., 2020). Such challenges associated with using neurotrophic factors to increase GAP-43 and BASP1 levels can be avoided by using other pharmacological agents that can cross the BBB and modulate their activities, for instance, via phosphorylation. A potential candidate is the FDA-approved CaN inhibitor Tacrolimus. In a rat model of PD, tacrolimus was shown to cross the BBB, alter phosphorylation of GAP-43 and BASP1, and confer neuroprotection at doses 10-fold lower than the standard immunosuppressive dose (Caraveo et al., 2017). At sub-immunosuppressive doses, the risk of secondary effects, such as opportunistic infections, posterior reversible leukoencephalopathy, and seizures typically achieved at clinical doses would be avoided. Moreover, these lower Tacrolimus doses would finely tune GAP-43 and BASP1 to drive sufficient, but not hyperactive, axonal sprouting in a spatially and temporally confined manner. This regulation is necessary to promote regeneration specifically in the affected neuronal populations at the right time window while preventing potential complications arising from hyperconnectivity. Additionally, external inhibitory factors in the central nervous system (Fournier and Strittmatter, 2001) and other disease-associated factors (Phinney et al., 1999) need to be prevented from blocking axon growth or driving the formation of aberrant connections. Moreover, determining the stages of pathogenesis at which regeneration of remaining axons can be protective will be important. Further exploration of these areas will facilitate the development of GAP-43- and BASP1-targeting therapies for neurodegenerative diseases.

## Author Contributions

DC contributed by writing and revising the entire review, as well as generating figures. AS contributed to writing the section “Involvement in Neural Injury Response” and building Figure 1. GC conceived the review, contributed to the concluding remarks, and edited the review. All authors contributed to the article and approved the submitted version.

## Conflict of Interest

The authors declare that the research was conducted in the absence of any commercial or financial relationships that could be construed as a potential conflict of interest.
